# Supplementary material for: Serum Levels of Myonectin Are Lower in Adults with Metabolic Syndrome and Are Negatively Correlated with Android Fat Mass
Source: Int J Mol Sci. 2023 Apr 7;24(8):6874. doi: 10.3390/ijms24086874 (PMC10138930; doi:10.3390/ijms24086874)
Supplement: Supplementary file 1 [file ijms-24-06874-s001.zip › ijms-2226240-supplementary.pdf]

International Journal of
Molecular Sciences

Article

Serum Levels of Myonectin Are Lower in Adults with Metabolic Syndrome and Are Negatively Correlated with Android Fat Mass

Jorge L. Petro [1,2,*], María Carolina Fragozo-Ramos [1], Andrés F. Milán [1], Juan C. Aristizabal [1], Jaime A. Gallo-Villegas [3], Juan C. Calderón [1,*]

[1] Physiology and Biochemistry Research Group—PHYSIS, Faculty of Medicine, University of Antioquia, Medellín 050010, Colombia
2 Research Group in Physical Activity, Sports and Health Sciences—GICAFS, Universidad de Córdoba, Montería 230002, Colombia
3 Sports Medicine Postgraduate Program, and GRINMADE Research Group, SICOR Center, Faculty of Medicine, University of Antioquia, Medellín 050010, Colombia

*Correspondence: jorge.petro@udea.edu.co (J.L.P.); jcalderonv00@yahoo.com (J.C.C.)

Supplementary Materials

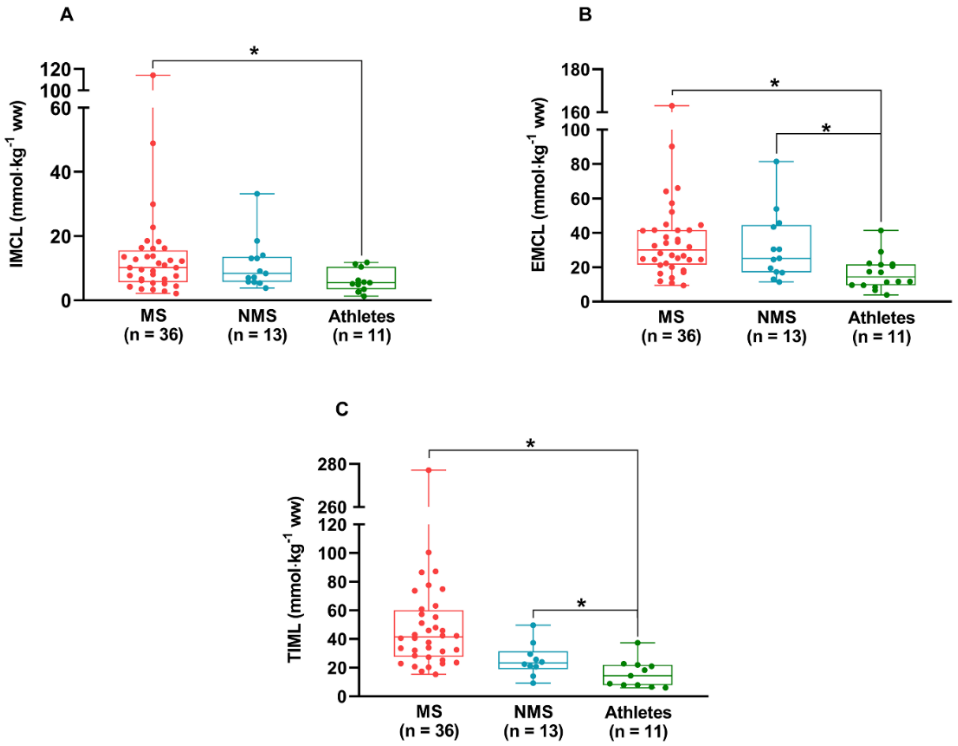

Figure S1. ^{1}H-MRS allows to demonstrate higher intramuscular lipid content in adults with metabolic risk compared with healthy controls. A. IMCL, Intramyocellular lipid content; B. EMCL, Extramyocellular lipid content; C. TIML, Total intramuscular lipid content (IMCL +

EMCL). Characteristics of the groups (mean±standard deviation or median (interquartile range): MS, metabolic syndrome (age: 51.0 (46.0–56.0) years, female sex: 70.1%, BMI: 30.6±4.0 kg·m^{-2}); NMS, no metabolic syndrome (age: 53.0 (45.5–57.5) years, female: 72.4%, BMI: 27.0±3.5 kg·m^{-2}); Athletes (age: 19.0±1.9 years, females: 81.8%, BMI: 24.6±2.1 kg·m^{-2}); *, statistically significant difference (P<0.05), comparisons between the groups made with the Kruskal-Wallis test. ^{1}H-MRS, proton nuclear magnetic resonance spectroscopy.

Table S1. Multiple linear regression model showing the relationship between myonectin and Android/gynoid fat mass ratio, excluding subjects who were treated with cholesterol medications.

	**β[a]**	**t**	**P-value**	**95% CI[b]**	**R^2**	**adj. R^2**	**P-value - F**	**D-W**
**And/Gyn**								
Constant	0.315	1.36	0.178	-0.147 to 0.777				
Myonectin, ng·mL^{-1}	-0.010	-2.242	0.028	-0.019 to -0.001				
Age, years	-0.300	-5.142	<0.001	-0.416 to -0.184	0.532	0.501	<0.001	2.113
Female sex	0.006	2.428	0.018	0.001 to 0.011				
FMI, kg·m^{-2}	0.018	2.641	0.010	0.004 to 0.031				
LMI, kg·m^{-2}	0.001	0.090	0.928	-0.017 to 0.019				

The analysis was performed with n=80; [a] non-standardized coefficient (indicates the average change that corresponds to the dependent variable for each unit of change in the independent variable); [b] 95% confidence interval for β; D-W, Durbin Watson test. And/Gyn, Android/gynoid fat mass ratio; FMI, fat mass index; LMI, lean mass index.
